# Supplementary material for: ZSWIM8 destabilizes many murine microRNAs and is required for proper embryonic growth and development
Source: Genome Res. 2023 Sep;33(9):1482–96. doi: 10.1101/gr.278073.123 (PMC10620050; doi:10.1101/gr.278073.123)
Supplement: Supplement 5 [file Supplementary_Materials.pdf]

Extended Methods

Bulk RNA-seq

Transcripts per million (TPM) values were computed by dividing the counts mapping to each gene by the length of its longest annotated mRNA isoform, and then dividing by the sum of this measure across all genes, and multiplying by 10^6. Counts arising from *Xist* or *Tsix* were used to assign sex (one *Zswim8*$^{+/–}$ embryo was female; the other *Zswim8*$^{+/–}$ embryo and two *Zswim8*$^{–/–}$ embryos were male), and removed before proceeding with analysis. No substantial differences in ZSWIM8 sensitivity of miRNAs were observed between tissues of male and female embryos (Fig. S3G).

sRNA-seq

sRNA-seq was not performed using tissues from *Zswim8*$^{+/+}$ E18.5 embryos, as they were not always present in the same litters as *Zswim8*$^{–/–}$ embryos. However, *Zswim8*$^{+/+}$ littermates were present in an E14.5 litter from which sRNA-seq libraries were prepared using RNA extracted from whole embryos. Here, the $\log_2$-fold changes in miRNAs of *Zswim8*$^{–/–}$embryos calculated using either *Zswim8*$^{+/+}$ or *Zswim8*$^{+/–}$ embryos as the reference group were highly correlated (Figure S3H). Moreover, the ZSWIM8-sensitive miRNAs had an additional 1.2-fold increase in $\log_2$-fold changes when using *Zswim8*$^{+/+}$ as the reference, compared to *Zswim8*$^{+/–}$ (Figure S3H), which indicated a slight, but detectable, reduction of ZSWIM8 function in the *Zswim8*$^{+/–}$ embryos. If this reduction also occurs at E18.5, then the use of the results from *Zswim8*$^{+/–}$ animals (rather than *Zswim8*$^{–/–}$ animals) as the reference presumably led us to slightly underestimate ZSWIM8 sensitivities in this study.

As noted in the Methods section, miRNAs were quantified by string-matching to dictionaries. For annotated miRNAs, a dictionary was generated from mature miRNA sequences whose first 19 nt were unique, in order to avoid ambiguities caused by 3′ alterations, as previously described (Shi et al., 2020). For the 48 annotated miRNAs whose first 19 nt were not unique, all miRNAs sharing a 19-nt prefix were collapsed to create a single dictionary entry with sequence of the 19-nt prefix, and given a name that combined the names of the collapsed miRNAs (e.g., mmu-miR-466j/mmu-miR-466m-5p/mmu-miR-669m-5p). This procedure added an additional 21 entries to the dictionary. Counting of 5′ miRNAs isoforms was performed similarly as for annotated miRNAs, except that the miRNA dictionary was expanded to include sequences of all 1- and 2-nt 5′ truncations, and all 1- and 2-nt 5′ extensions of annotated miRNAs potentially arising from processing of annotated precursors.

Depth-normalization (counts per million, CPM) was used for all analyses of sRNA-seq data. An alternative normalization to the spike-in oligos added to each library did not produce substantial differences, indicating negligible changes in absolute abundance of bulk miRNA levels between *Zswim8* genotypes of the same tissue. Thus, CPM normalization was chosen to avoid potential experimental variability that might arise from RNA quantification and addition of the spike-in oligos.

ZSWIM8-senstive miRNAs were called as detailed in the Methods section. In a few cases (miR-335-3p, miR-429-5p, miR-466i-3p, miR-544-3p, miR-652-5p, miR-764-5p, miR-497a-3p, miR-744-3p, miR-99b-3p), the annotated passenger strand was ZSWIM8-sensitive, in which case, for purposes of our analyses, the ZSWIM8-sensitive strand was considered the guide strand, and the other strand was considered the passenger strand. As described in the Methods section, a significantly up-regulated miRNA produced from a single locus was classified as a ZSWIM8-sensitive miRNA if its $\log_2$ fold change upon loss of ZSWIM8 was significantly greater than that of its passenger strand, as would be expected of substrates of TDMD under the assumption that the change in the level of the miRNA is affected by ZSWIM8-mediated degradation, but not change in its production rate, nor the production or degradation rates of the passenger. In the case of a significantly up-regulated miRNA produced from more than one locus, the analogous assumptions led to classification of the

miRNA as a ZSWIM8-sensitive miRNA if its $\log_2$ fold change upon *Zswim8* loss was significantly greater than the $\log_2$ of the sum of its passenger strands' levels in *Zswim8*$^{-/-}$ subtracted by the $\log_2$ of the sum of its passenger strands' levels in *Zswim8*$^{+/-}$. An additional simplifying assumption was that the production rates of the miRNA and passenger produced from a locus differed by the same multiplicative factor for all loci producing the miRNA. For these analyses, the aforementioned 'significantly greater than' condition was achieved if the two compared quantities were separated beyond their standard errors, as calculated by DESeq2 (1.26.0) (Love et al., 2014) or propagated therefrom.

As noted in the Methods section, a miRNA was also called as ZSWIM8-sensitive if it showed increases in at least 11 of 12 *Zswim8*$^{-/-}$ tissues, with a median $\log_2$ fold-change > 0.2 across tissues, as well as a greater increase compared to that of its cognate strand, with a median difference in $\log_2$ fold-change > 0.2 across tissues. To estimate an upper limit on the false-discovery rate using these alternative criteria, we started with a simple model. When taking the miRNAs not called as ZSWIM8-sensitive as a background distribution for a binomial model of the probability that a miRNA's $\log_2$ fold change randomly happens to be greater than zero in at least 11 of 12 tissues, we found that a total of 1.26 miRNAs would be expected to meet this simple criterion by chance. Going beyond this simple model, our actual criteria were more stringent in two ways: (1) we additionally required a median miRNA $\log_2$ fold change > 0.2 (not > 0) relative to that of the passenger in 11 of 12 tissues, and (2) we additionally required a median $\log_2$ fold change > 0.2 in all tissues. Thus, the miRNA must increase consistently in *Zswim8*$^{-/-}$ tissues, and this increase must also consistently exceed that of its passenger strand, making the number of expected false discoveries much lower than the upper limit of 1.26 predicted by the less-stringent background model.

From the union of miRNAs annotated as ZSWIM8-sensitive across the twelve profiled tissues, six were manually removed and recategorized as secondarily ZSWIM8-sensitive (miR-743b-3p, miR-883a-3p, miR-883b-3p, miR-881-3p, miR-880-3p, and miR-742-3p). These six were likely erroneously categorized because they are members of a miRNA cluster that was likely transcriptionally up-regulated in placenta (Fig. S3E), but unlike the other detected miRNAs in the cluster, had passenger strands that did not meet our detection threshold. In addition, two (miR-451a and miR-122-5p) were removed on the basis of possible contamination with small quantities of material from blood or liver during dissection, and two more (miR-429-3p and miR-135b-5p) were removed because they were down-regulated in many tissues except for one, and were thus possibly misannotated by our criteria by chance. miRNAs classified as ZSWIM8-sensitive were examined within individual replicate libraries to ensure that classification did not appear to be driven by sex differences between the samples (Fig. S3G).

For arm-switching analysis, four ZSWIM8-sensitive miRNAs were each annotated as encoded by two separate loci (miR-92a-3p, miR-194-5p, miR-7-5p, and miR-29b-3p), and each of these eight hairpins was counted as a ZSWIM8-sensitive miRNA hairpin locus.

Supplemental Figures

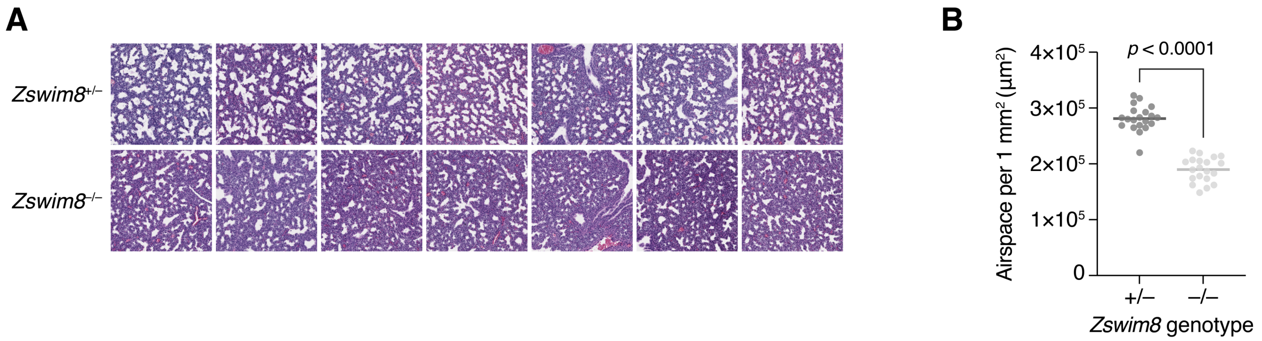

Figure S1. Airspace defect in *Zswim8*$^{-/-}$ embryonic lung; related to Fig. 2. (A) Images of representative H&E-stained fixed sections from lungs of a E18.5 *Zswim8*$^{+/-}$ embryo and a *Zswim8*$^{-/-}$ littermate. (B) Quantification of airspace area from 20 sections with adjacent sections spaced >200 µm apart. *p*-value from Mann-Whitney *U* test.

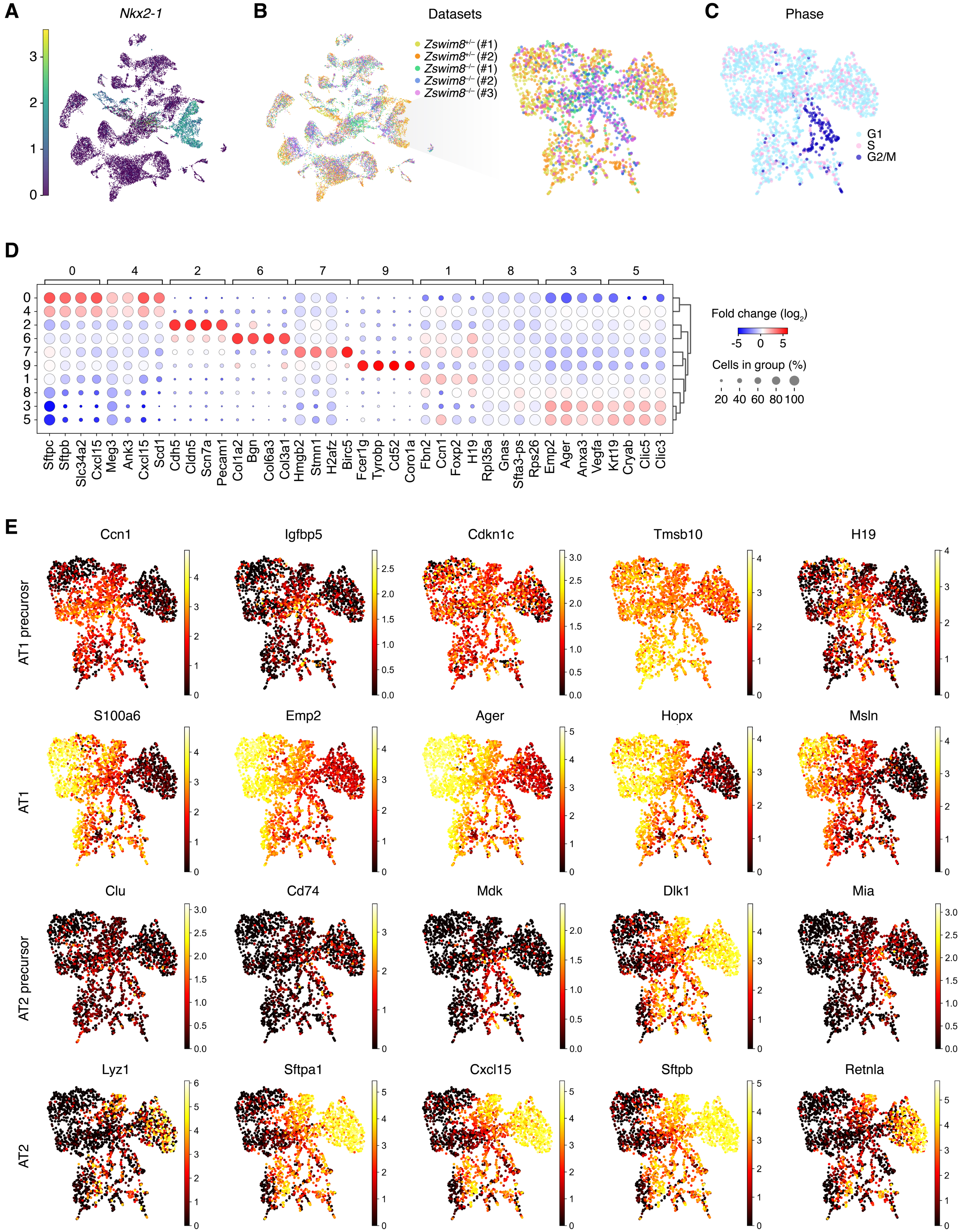
A
Nkx2-1
B
Datasets
Zswim8+/− (#1)
Zswim8+/− (#2)
Zswim8−/− (#1)
Zswim8−/− (#2)
Zswim8−/− (#3)
C
Phase
G1
S
G2/M
D
0
4
2
6
7
9
1
8
3
5
Fold change (log2)
-5 0 5
Cells in group (%)
20 40 60 80 100
Sftpc
Sftpb
Slc34a2
Cxcl15
Meg3
Ank3
Cxcl15
Scd1
Cdh5
Cldn5
Scn7a
Pecam1
Col1a2
Bgn
Col6a3
Col3a1
Hmgb2
Stmn1
H2afz
Birc5
Fcer1g
Tyrobp
Cd52
Coro1a
Fbn2
Ccn1
Foxp2
H19
Rpl35a
Gnas
Sfta3-ps
Rps26
Emp2
Ager
Anxa3
Vegfa
Krt19
Cryab
Clic5
Clic3
E
AT1 precurosr
Ccn1
Igfbp5
Cdkn1c
Tmsb10
H19
AT1
S100a6
Emp2
Ager
Hopx
Msln
AT2 precursor
Clu
Cd74
Mdk
Dlk1
Mia
AT2
Lyz1
Sftpa1
Cxcl15
Sftpb
Retnla

Figure S2. Single-cell RNA-sequencing from lungs of *Zswim8*$^{-/-}$ embryos; related to Fig. 3. (A) The three most significantly enriched genes in each unsupervised cluster generated from all cells captured; related to Fig. 3A. Row labels indicate cluster number, and column labels indicate enriched genes grouped by cluster number. Rows are hierarchically clustered. Size of dots indicates the fraction of cells in given cluster with detectable counts for a given gene. Fill color of dots indicates the fold difference in mean expression in a given cluster, relative to the union of all other clusters; color bar is in units of $\log_2$. Significance determined by Wilcoxon rank-sum test, adjusted by the Benjamini-Hochberg method. (B) UMAP of all cells captured, as in Fig. 3A, with color indicating expression of the canonical lung epithelial marker *Nkx2-1*. Color bar is in units of log(1+ CP10K); related to Fig. 3A. (C) Left: As in Fig. 3A, UMAP of all cells captured, with color indicating individual dataset; related to Fig. 3A. Right: As in Fig. 3B, UMAP re-embedding of cells of Cluster 2 shown in Fig. 3A (corresponding largely to *Nkx2-1*-positive cells), with color indicating individual dataset; related to Fig. 3B. (D) As in Fig. 3B, UMAP re-embedding of cells of Cluster 2 shown in Fig. 3A (corresponding largely to *Nkx2-1*-positive cells), with color indicating inferred cell cycle phase; related to Fig. 3B. (E) The four most significantly enriched genes in each cluster generated from re-clustering of re-embedded of cells of Cluster 2 shown in Fig. 3A; related to Fig. 3B. (F) Expression of reported marker genes for AT1 precursor, AT1, AT2 precursor, and AT2 cells (Frank et al., 2019) in re-embedding of cells of Cluster 2 shown in Fig. 3A; related to Fig. 3B–C.

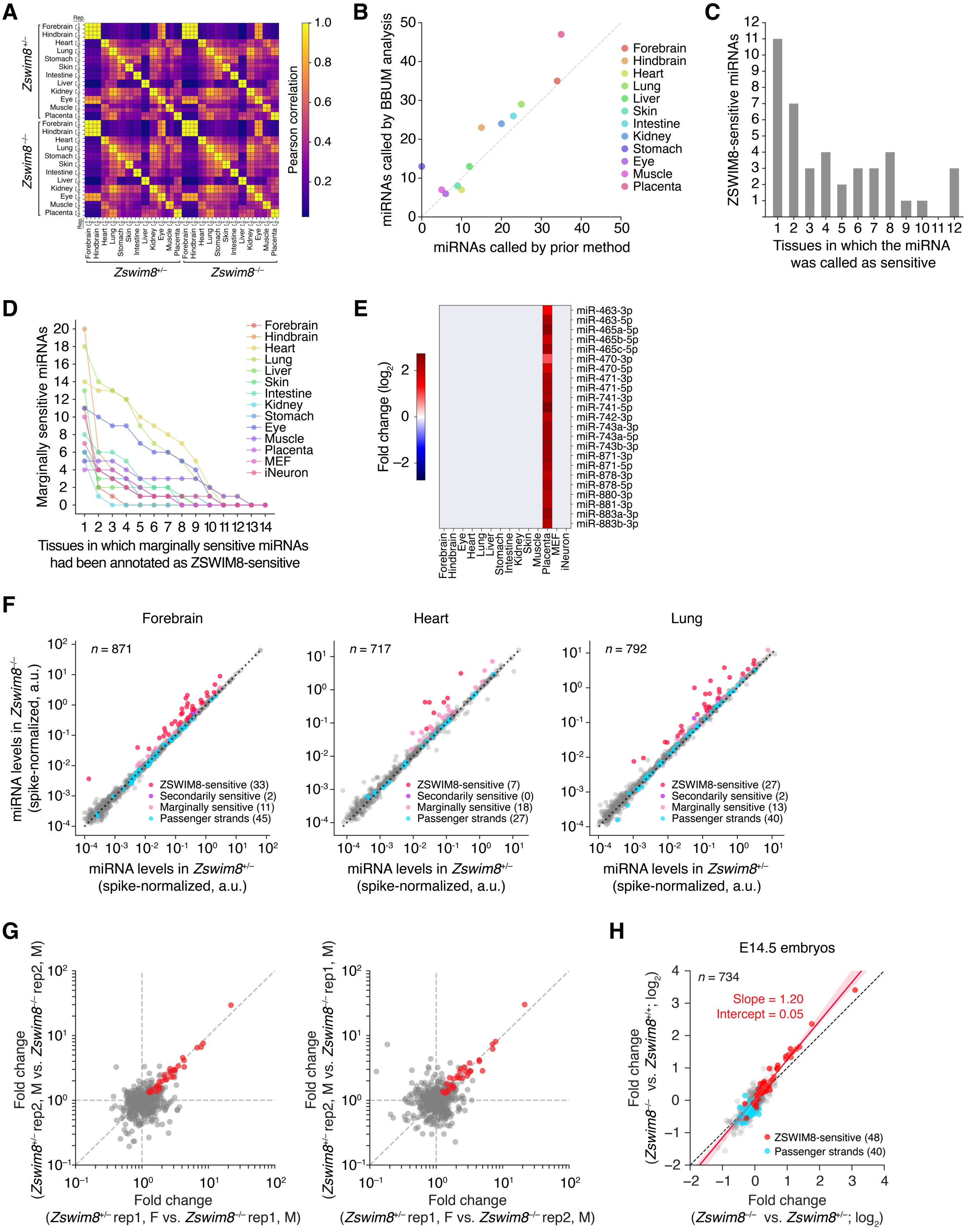
A
Rep.
Forebrain
Hindbrain
Heart
Lung
Stomach
Skin
Intestine
Liver
Kidney
Eye
Muscle
Placenta
Zswim8+/–
Zswim8–/–
Pearson correlation
1.0
0.8
0.6
0.4
0.2
B
miRNAs called by BBUM analysis
miRNAs called by prior method
Forebrain
Hindbrain
Heart
Lung
Liver
Skin
Intestine
Kidney
Stomach
Eye
Muscle
Placenta
C
ZSWIM8-sensitive miRNAs
Tissues in which the miRNA was called as sensitive
D
Marginally sensitive miRNAs
Tissues in which marginally sensitive miRNAs had been annotated as ZSWIM8-sensitive
Forebrain
Hindbrain
Heart
Lung
Liver
Skin
Intestine
Kidney
Stomach
Eye
Muscle
Placenta
MEF
iNeuron
E
Fold change (log2)
miR-463-3p
miR-463-5p
miR-465a-5p
miR-465b-5p
miR-465c-5p
miR-470-3p
miR-470-5p
miR-471-3p
miR-471-5p
miR-741-3p
miR-741-5p
miR-742-3p
miR-743a-3p
miR-743a-5p
miR-743b-3p
miR-871-3p
miR-871-5p
miR-878-3p
miR-878-5p
miR-880-3p
miR-881-3p
miR-883a-3p
miR-883b-3p
Forebrain
Hindbrain
Eye
Heart
Lung
Liver
Stomach
Intestine
Kidney
Skin
Muscle
Placenta
MEF
iNeuron
F
Forebrain
n = 871
ZSWIM8-sensitive (33)
Secondarily sensitive (2)
Marginally sensitive (11)
Passenger strands (45)
Heart
n = 717
ZSWIM8-sensitive (7)
Secondarily sensitive (0)
Marginally sensitive (18)
Passenger strands (27)
Lung
n = 792
ZSWIM8-sensitive (27)
Secondarily sensitive (2)
Marginally sensitive (13)
Passenger strands (40)
miRNA levels in Zswim8–/– (spike-normalized, a.u.)
miRNA levels in Zswim8+/– (spike-normalized, a.u.)
G
Fold change (Zswim8+/– rep2, M vs. Zswim8–/– rep2, M)
Fold change (Zswim8+/– rep1, F vs. Zswim8–/– rep1, M)
Fold change (Zswim8+/– rep2, M vs. Zswim8–/– rep1, M)
Fold change (Zswim8+/– rep1, F vs. Zswim8–/– rep2, M)
H
E14.5 embryos
n = 734
Slope = 1.20
Intercept = 0.05
ZSWIM8-sensitive (48)
Passenger strands (40)
Fold change (Zswim8–/– vs. Zswim8+/+; log2)
Fold change (Zswim8–/– vs. Zswim8+/–; log2)

Figure S3. Influence of ZSWIM8 on miRNAs across embryonic tissues; related to Fig. 4. (A) Correspondence of miRNA levels between E18.5 embryonic tissues of two biological replicates, as measured by sRNA-seq. Color map indicates Pearson correlation. (B) Number of significantly ZSWIM8-sensitive miRNAs in E18.5 embryonic tissues called by BBUM analysis (Wang and Bartel, 2023), compared to that called by our original method (Shi et al., 2020). (C) The number of miRNAs called as ZSWIM8-sensitive in E18.5 embryos by both significant upregulation in BBUM analysis and significant elevation of guide strand above passenger strand, compared to the number of tissues in which they were independently called as ZSWIM8-sensitive. (D) The frequency by which marginally sensitive miRNAs were independently called as ZSWIM8-sensitive in other tissues. Shown for each tissue are the number of marginally sensitive miRNAs plotted as a function of the number of other datasets (embryonic tissues, as well as MEF and iNeuron cells (Shi et al., 2020)) in which these miRNAs were independently called as ZSWIM8-sensitive. (E) Changes in levels of all detected members of an X-linked genomic cluster encoding miR-743a, miR-743b, miR-742, miR-883a, miR-883b, miR-471, miR-741, miR-463, miR-880, miR-878, miR-881, miR-871, miR-470, miR-465d, miR-465c, miR-465b, and miR-465a. Color bar indicates $\log_2$ fold change; gray, not detected. Further supporting the conclusion that this cluster is transcriptionally up-regulated in *Zswim8*$^{-/-}$ embryos, analysis of RNA-seq data from *Zswim8*$^{-/-}$ placenta (GEO accession GSE231447) showed an elevated number of normalized reads mapping to this cluster. (F) Levels of miRNAs in three representative tissues from *Zswim8*$^{+/-}$ and *Zswim8*$^{-/-}$ E18.5 embryos, as measured by sRNA-seq normalized to internal spike-in standards (a.u., arbitrary units). Colored points correspond to those shown in Figure 4. (G) Correspondence of ZSWIM8 sensitivity of miRNAs in forebrain between individual samples derived from male (M) and female (F) embryos of two biological replicates, as measured by sRNA-seq. Shown are comparisons of fold changes in miRNA levels between samples of the indicated sexes and genotypes. miRNAs called as ZSWIM8-sensitive in forebrain are shown in red. Similar results (not shown) were observed in the eleven other embryonic tissues. (H) Similar, albeit slightly greater, ZSWIM8 sensitivity observed when comparing to results from *Zswim8*$^{+/+}$ embryos, rather than *Zswim8*$^{+/-}$ embryos. Plotted are miRNA fold changes in *Zswim8*$^{-/-}$ E14.5 embryos, as measured by sRNA-seq, comparing to levels observed in either *Zswim8*$^{+/+}$ or *Zswim8*$^{+/-}$ embryos. Points for ZSWIM8-sensitive miRNAs identified in at least one E18.5 tissue are colored red, and those of their passenger strands are colored blue. A linear model fit to the ZSWIM8-sensitive miRNAs is shown in red (shading indicates the 95% confidence interval), with slope and intercept.

A

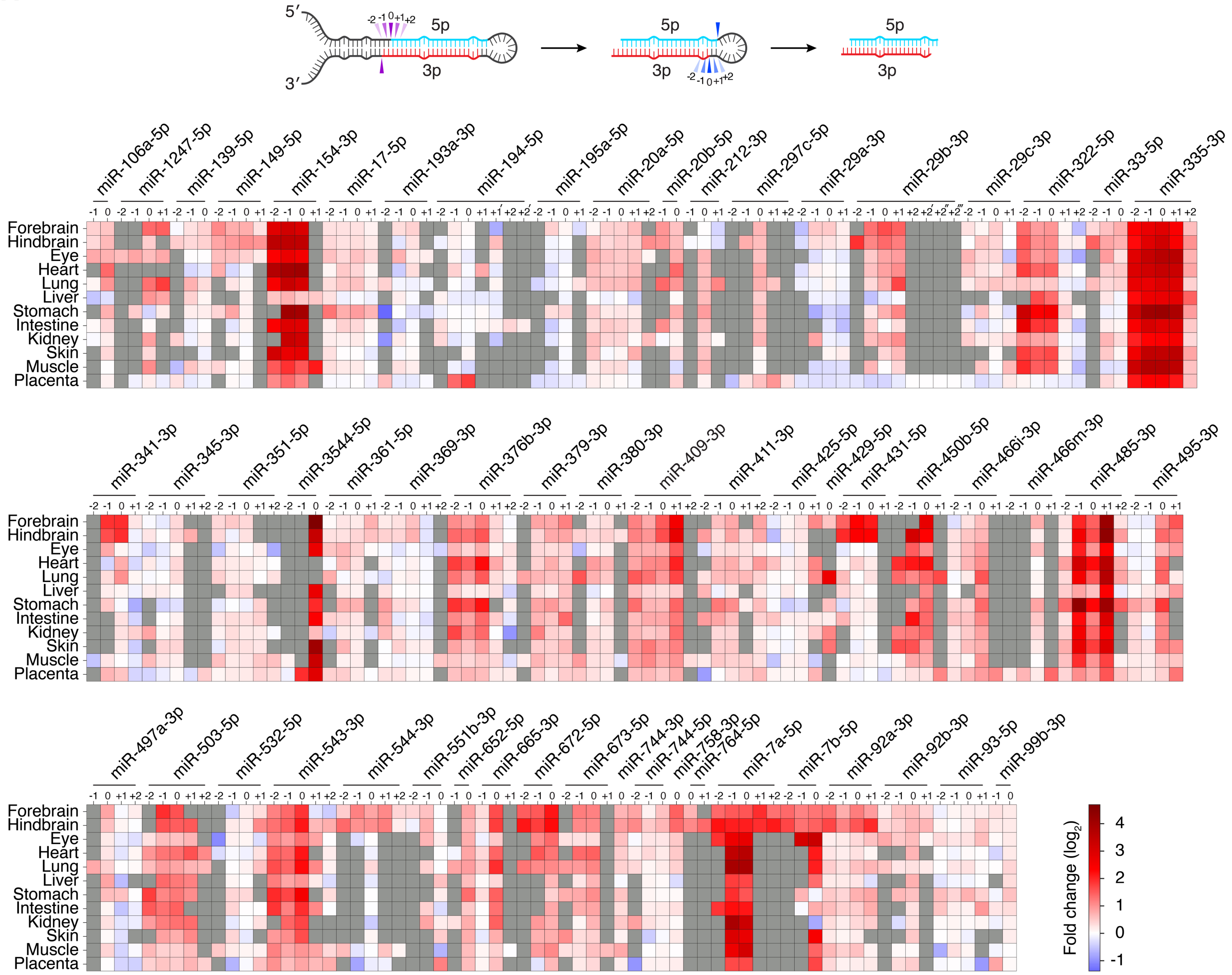

5′
3′
5p
3p
-2 -1 0 +1 +2
miR-106a-5p
miR-1247-5p
miR-139-5p
miR-149-5p
miR-154-3p
miR-17-5p
miR-193a-3p
miR-194-5p
miR-195a-5p
miR-20a-5p
miR-20b-5p
miR-212-3p
miR-297c-5p
miR-29a-3p
miR-29b-3p
miR-29c-3p
miR-322-5p
miR-33-5p
miR-335-3p
miR-341-3p
miR-345-3p
miR-351-5p
miR-3544-5p
miR-361-5p
miR-369-3p
miR-376b-3p
miR-379-3p
miR-380-3p
miR-409-3p
miR-411-3p
miR-425-5p
miR-429-5p
miR-431-5p
miR-450b-5p
miR-466i-3p
miR-466m-3p
miR-485-3p
miR-495-3p
miR-497a-3p
miR-503-5p
miR-532-5p
miR-543-3p
miR-544-3p
miR-551b-3p
miR-652-5p
miR-665-3p
miR-672-5p
miR-673-5p
miR-744-3p
miR-744-5p
miR-758-3p
miR-764-5p
miR-7a-5p
miR-7b-5p
miR-92a-3p
miR-92b-3p
miR-93-5p
miR-99b-3p
Forebrain
Hindbrain
Eye
Heart
Lung
Liver
Stomach
Intestine
Kidney
Skin
Muscle
Placenta
Fold change (log_2)
4
3
2
1
0
-1

B

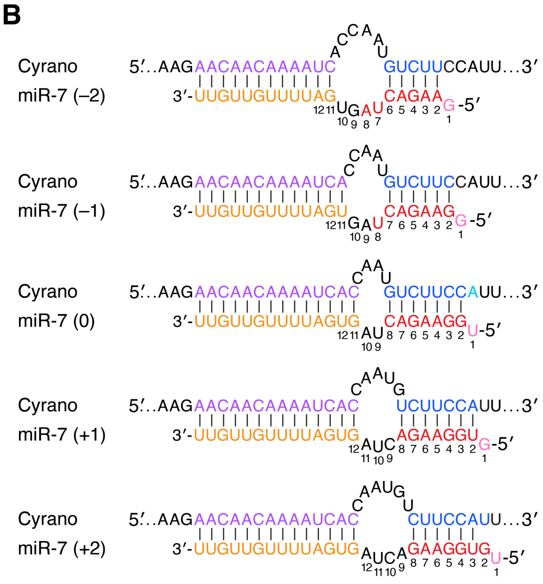

Cyrano
5′...AAGAACAACAAAAUC ACCAAU GUCUUCCAUU...3′
miR-7 (–2)
3′-UUGUUGUUUUAG UGAU CAGAAG-5′
Cyrano
5′...AAGAACAACAAAAUCA CAAU GUCUUCCAUU...3′
miR-7 (–1)
3′-UUGUUGUUUUAGU GAU CAGAAGG-5′
Cyrano
5′...AAGAACAACAAAAUCAC CAAU GUCUUCCAUU...3′
miR-7 (0)
3′-UUGUUGUUUUAGUG AU CAGAAGGU-5′
Cyrano
5′...AAGAACAACAAAAUCAC CAAUG UCUUCCAUU...3′
miR-7 (+1)
3′-UUGUUGUUUUAGUG AUC AGAAGGUG-5′
Cyrano
5′...AAGAACAACAAAAUCAC CAAUGU CUUCCAUU...3′
miR-7 (+2)
3′-UUGUUGUUUUAGUG AUCA GAAGGUGU-5′

Figure S4. Influence of ZSWIM8 on miRNAs Isoforms Across Embryonic Tissues; related to Fig. 4.
(A) The influence of ZSWIM8 on 5′ isoforms of miRNAs classified as ZSWIM8-sensitive in at least one of the twelve embryonic tissues. The heatmap indicates fold-changes (key) observed when comparing the results for tissues from *Zswim8*$^{–/–}$ E18.5 embryos with those from *Zswim8*$^{+/–}$ E18.5 embryos. Gray squares indicate contexts in which the number of miRNA reads did not exceed the detection threshold of 5 CPM in each library prepared from the corresponding tissue, as in Figure 5B. –1, one nt 5′ truncation isoform; –2, two nt 5′ truncation isoform; 0, annotated isoform; +1, one nt 5′ extension isoform; +2, two nt 5′ extension isoform of the indicated miRNA. For miRNAs arising from more than one precursor, multiple unique extension isoforms may be possible; these are indicated by ′, ″ , etc. (B) Representations of possible pairing configurations between Cyrano and 5′ isoforms of miR-7a-5p.

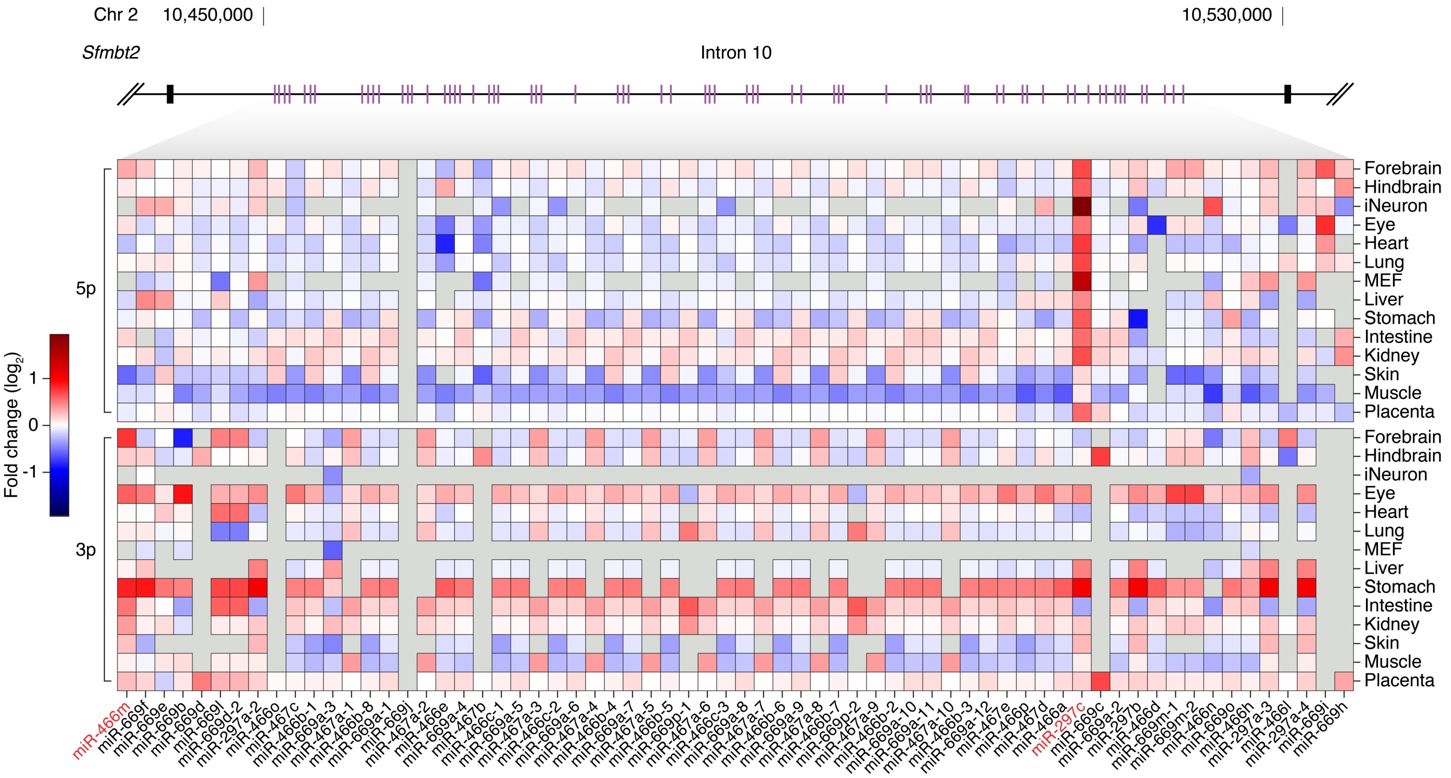

Figure S5. Influence of ZSWIM8 on expression of miRNAs of the rodent-specific cluster in intron 10 of the *Sfmbt2* gene; related to Fig. 6. Shown are fold changes in miRNA levels observed in *Zswim8*$^{-/-}$ tissues, relative to *Zswim8*$^{+/-}$ tissues from E18.5 embryos, as well as in MEFs and iNeurons (Shi et al., 2020). The miRNAs are ordered by genomic position within the cluster, and each value is the average of two biological replicates, as in Figure 6. Red text denotes ZSWIM8-sensitive miRNAs. As described in the Extended Methods section, many of the paralogous miRNAs within this cluster have the same first 19 nt, and are thus combined into a single entry for mapping and quantification.

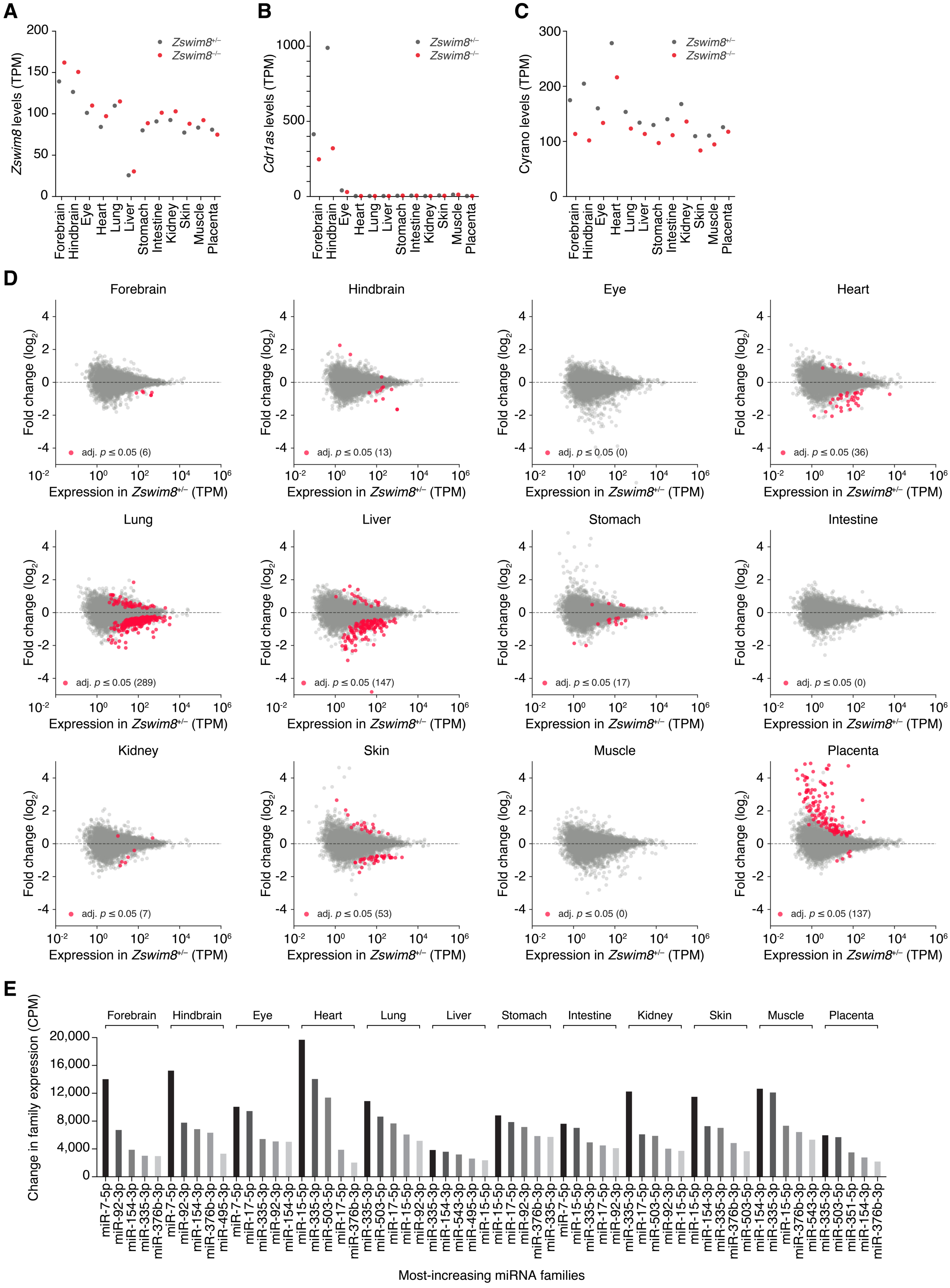

A
Zswim8 levels (TPM)
Zswim8+/–
Zswim8–/–
B
Cdr1as levels (TPM)
C
Cyrano levels (TPM)
Forebrain
Hindbrain
Eye
Heart
Lung
Liver
Stomach
Intestine
Kidney
Skin
Muscle
Placenta
D
Fold change (log2)
Expression in Zswim8+/– (TPM)
adj. p ≤ 0.05 (6)
adj. p ≤ 0.05 (13)
adj. p ≤ 0.05 (0)
adj. p ≤ 0.05 (36)
adj. p ≤ 0.05 (289)
adj. p ≤ 0.05 (147)
adj. p ≤ 0.05 (17)
adj. p ≤ 0.05 (0)
adj. p ≤ 0.05 (7)
adj. p ≤ 0.05 (53)
adj. p ≤ 0.05 (0)
adj. p ≤ 0.05 (137)
E
Change in family expression (CPM)
Most-increasing miRNA families
Forebrain: miR-7-5p, miR-92-3p, miR-154-3p, miR-335-3p, miR-376b-3p
Hindbrain: miR-7-5p, miR-92-3p, miR-154-3p, miR-376b-3p, miR-495-3p
Eye: miR-7-5p, miR-17-5p, miR-335-3p, miR-92-3p, miR-154-3p
Heart: miR-15-5p, miR-335-3p, miR-503-5p, miR-17-5p, miR-376b-3p
Lung: miR-335-3p, miR-503-5p, miR-17-5p, miR-15-5p, miR-92-3p
Liver: miR-335-3p, miR-154-3p, miR-543-3p, miR-495-3p, miR-15-5p
Stomach: miR-15-5p, miR-17-5p, miR-92-3p, miR-376b-3p, miR-335-3p
Intestine: miR-7-5p, miR-15-5p, miR-335-3p, miR-17-5p, miR-92-3p
Kidney: miR-335-3p, miR-17-5p, miR-503-5p, miR-92-3p, miR-15-5p
Skin: miR-15-5p, miR-154-3p, miR-335-3p, miR-376b-3p, miR-503-5p
Muscle: miR-154-3p, miR-335-3p, miR-15-5p, miR-376b-3p, miR-543-3p
Placenta: miR-335-3p, miR-503-5p, miR-351-5p, miR-154-3p, miR-376b-3p

Figure S6. Influence of ZSWIM8 on levels of miRNA targets across embryonic tissues; related to Fig. 7. (A) Expression of *Zswim8* tissues of *Zswim8*$^{+/-}$ and *Zswim8*$^{-/-}$ E18.5 embryos across two biological replicates, as quantified by RNA-seq. TPM; transcripts per million. (B) Expression of the circular RNA *Cdr1as* in tissues of *Zswim8*$^{+/-}$ and *Zswim8*$^{-/-}$ E18.5 embryos. (C) Expression of the long noncoding RNA Cyrano in tissues of *Zswim8*$^{+/-}$ and *Zswim8*$^{-/-}$ E18.5 embryos. (D) Expression levels of RNA species in tissues of E18.5 embryos, as quantified by RNA-seq. Shown are fold changes in levels of *Zswim8*$^{-/-}$ tissues, relative to *Zswim8*$^{+/-}$. Highlighted are differentially expressed species with adjusted p-value < 0.05 as computed by DESeq2 (Love et al., 2014). (E) Changes in the levels of miRNA seed-family members, as quantified by sRNA-seq. Shown are the five most-increasing families, as defined by total changes in depth-normalized counts per million (CPM), in *Zswim8*$^{-/-}$ E18.5 tissues, relative to *Zswim8*$^{+/-}$; related to Fig. 7B.
